# Stress Distribution Analysis of Threaded Implants for Digital Dentistry

**DOI:** 10.3390/ijerph191912674

**Published:** 2022-10-04

**Authors:** Seokho Ahn, Jaesung Kim, Seok Chan Jeong, Myungil Kim, Cheolyoung Kim, Dongki Park

**Affiliations:** 1 Department of Digital Manufacturing, Hanbat National University, 125 Dongseo-daero, Yuseong-gu, Daejeon 34158, Korea; 2 Department of Industry-Academic Convergence, Hanbat National University, 125 Dongseo-daero, Yuseong-gu, Daejeon 34158, Korea; 3 Department of e-Business, Dong-Eui University, Busanjin-gu, Busan 47340, Korea; 4 Div. of National Supercomputing Intelligent Simulation Center, Korea Institute of Science and Technology Information 245, Daehak-ro, Yuseong-gu, Daejeon 34141, Korea; 5 Implant Research Laboratory, Cybermed 6-26, Yuseong-daro 1205 beon-gil, Yuseong-gu, Daejeon 34104, Korea

**Keywords:** ANSYS mechanical, implant length, implant thickness, bone material properties, cortical bone, computer-aided engineering

## Abstract

In this study, stability evaluation is performed through structural analysis based on digital dental implant design variables. The design variables include the implant length and thickness, cortical bone thickness, and elastic modulus of the cancellous bone. Subsequently, the stress in the external cortical bone, in which numerous nerves exist, is analyzed. Results show that stress increases as the implant length decreases. However, when the implant length is 10 mm, the stress decreases, owing to stress dispersion at the lower section of the implant. Moreover, as the implant thickness increases, the stress decreases. As the elastic modulus of the cancellous bone decreases, the stress exerted on the cancellous bone decreases; consequently, the stress exerted on the cortical bone increases. Finally, as the thickness of the cortical bone increases, the stress decreases when a vertical load is applied. However, when a load is applied in the oblique direction, the stress increases. Based on data obtained via digital radiography, which is a digital dental technology, a more precise implantation plan will be established by substituting the data via structural analysis.

## 1. Introduction

Digital healthcare is a broad multidisciplinary concept that includes concepts at the intersection of digital technology and healthcare. Digital health transforms healthcare digitally by integrating software, hardware, and services. Digital health includes mobile health applications, electronic health records, wearable devices, telehealth and telemedicine, and personalized healthcare. The use of artificial intelligence (AI) in healthcare applications can enhance human decision making by automating and accelerating labor-intensive tasks. For example, many hospitals use AI-powered patient-monitoring tools to treat patients based on real-time reports. Using AI in medical imaging can reduce the number of clicks required to perform a task and determine the next step. Another AI application, digital twins, can be used to model medical devices and patients, as well as visualize the mechanism by which the devices operate in the real world [1,2].

The field of dentistry is actively integrating digital methods to keep pace with the development of various technologies. This approach, called digital dentistry, digitizes all information related to the patient’s oral cavity to optimize treatment for the patient’s oral condition. Digital radiography, electronic prescribing, computerized case presentations, digital-based surgical guides, and imaging and digital impressions for implant placement are among the digital technologies available in dentistry. Dentists hope to integrate proven digital technologies into dental care to deliver cutting-edge dental care that can be performed more efficiently, effectively, and comfortably. Digital dental technology allows consultations with patients and collaborations with other dentists to be performed faster, sooner, and more comprehensively. Furthermore, quality of care can be enhanced through improved diagnostics and precision restoration [3,4].

Among dental technologies, dental implants provide patient-specific care via the insertion of a metal or polymer-based product into the jawbone. This product is typically fabricated using titanium or zirconium, which increases the bone adhesion between the jawbone and the implant surface. The global dental implant and prosthetics market is expected to increase by 6.5 percent annually, i.e., from USD 9.56 billion in 2018 to USD 13.4 billion in 2023. Meanwhile, the dental implant market is expected to increase from USD 4.41 billion in 2019 at an annual average increase of 5.6 percent, thereby reaching USD 5.8 billion by 2024 [5].

The most commonly used implants include Branemark, Astra, and Osstem. Branemark is an implant using titanium that does not cause inflammation in soft tissues; long-term clinical data show that it can be used without rejection. Astra is an effective implant for bone with poor bone quality, owing to its excellent ability to maintain alveolar bone and low rate of bone loss. Osstem is an implant with excellent bone-implant fusion and initial fixation. There are pros and cons to each implant, but there is no significant difference; their applicability varies from patient to patient [6]. Dental implants manufactured by the three companies typically include root form implants consisting of cylindrical dental implants, straight screw dental implants, and conical screw dental implants. Among them, conical screw implants are most commonly used because they have excellent stability [7].

During implantation, a skilled dentist must directly establish a plan that considers the location and angle of implantation, which is labor intensive. Therefore, a technique must be devised to reduce the dependence on proficiency by providing real-time, physical, and visual guidance to doctors via locating suitable implant positions and selecting the necessary implants [8]. This can be accomplished the most easily using mobile applications. Data that can be entered into a model are a reduced-order model with sensitivity and immediate processing. Therefore, basic data analysis is required [9,10,11].

When selecting implants, their stability must be verified through stress structure analysis while considering the tooth grip force applied after implantation and the masticatory force that occurs during chewing. The most accurate method is to directly model human bones and implants, and then obtain the results after testing. However, the length, diameter, and placement angle of implants vary significantly, whereas the composition and physical properties of the cartilage vary by person. The modeling and evaluation of all these properties cannot be performed physically and in a timely manner. Therefore, a bone-implant model should be created using computer-aided engineering, and each variable must be interpreted to verify its stability.

In this regard, J. M. Jung [12] investigated the effects of stress on surrounding bones based on the shape of a neck implant. According to Jung, the neck shape affects the bone stress, i.e., a more curved neck results in a depression closer to the body, which consequently reduces the stress.

H. J. Jeong [13] investigated the effects of implant arrangement and load direction and confirmed that torsional alignment did not significantly affect the stress on an implant and alveolar bone; however, the number of implants significantly affected the stress magnitude.

Helder Oliveria [14] investigated the design of implants while considering strain and stress distributions under non-axial loading and reported that the stress distribution was affected by the implant shape, cortical bone thickness, and cancellous bone density. However, the deformation tendency has not yet been investigated.

**Figure 1:**
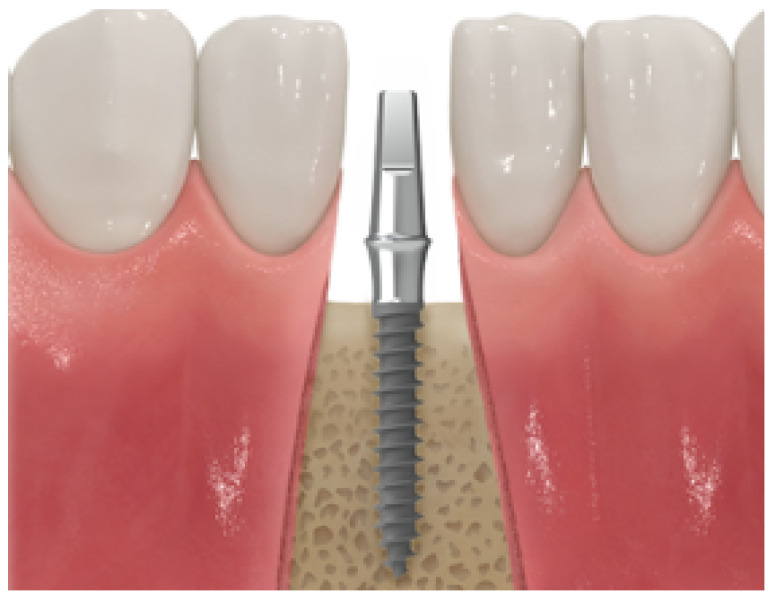
MACRO thread implant.

The medical community has conducted stress verification while considering various implant variables [15]; however, thorough verification analyses based on the cancellous bone elastic modulus and cortical bone thickness, which change depending on age, sex, and tooth position, remain insufficient. In addition, because the analyses were conducted separately based on each design variable, only the effect of each design variable on implants and cortical bones were investigated. Therefore, in this study, an analysis was conducted using four design variables: the implant length, thickness, cortical bone thickness, and cancellous bone elastic modulus. In addition, the effect of each parameter was investigated by considering the change in the implant thickness based on the change in the remaining design variables. Subsequently, implants used extensively in the real world are modeled three-dimensionally using ANSYS. The model used for the analysis assumed a state in which the crown was removed from an implant consisting of a crown, abutment, and screw, and it was assumed that the abutment and the screw were completely combined. As shown in Figure 1, a MACRO-type implant in which the threads are equally spaced was used, and 100 N dental and chewing forces were applied at room temperature.

Subsequently, we compared the effects of implant placement on the human body using the stress values obtained from this structural analysis. In the future, digitally captured three-dimensional (3D) data will be used to provide mobile consultation to patients and enable immediate collaboration.

## 2. Analysis Conditions and Method

The process for implant placement consists of planning, fixture placement, stabilization, abutment attachment, and crown fabrication and installation. Using the patient’s data obtained through X-ray imaging, a hole is drilled in the bone to place the implant. Then, after 3 to 4 months of bonding the bone and the fixture, the abutment is attached and the surgical site is healed, and finally the crown is manufactured and combined. In this study, the stability of dental implants combined through this process was compared through structural analysis to verify the stress resulting from the dental jaw force generated by chewing food.

### 2.1. Analysis Conditions

#### 2.1.1. 3D Model

**Figure 2:**
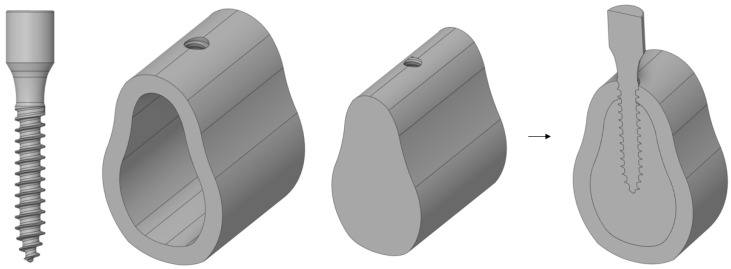
The components of the implant body.

Figure 2 shows a basic 3D model of the implants, cortical bone, and cancellous bone. The thread length and thickness of this implant were 13 and 2.5 mm, respectively. The cortical bone height, width, and thickness were 21, 15, and 2 mm, respectively. The cancellous bone was modeled to fill the cortical bone cavity completely. After the implant was placed and the bone and the fixture were completely combined, a hole was made in the cortical bone and cancellous bone with the same size as the screw thread so that they were completely combined.

#### 2.1.2. Boundary Condition

**Figure 3:**
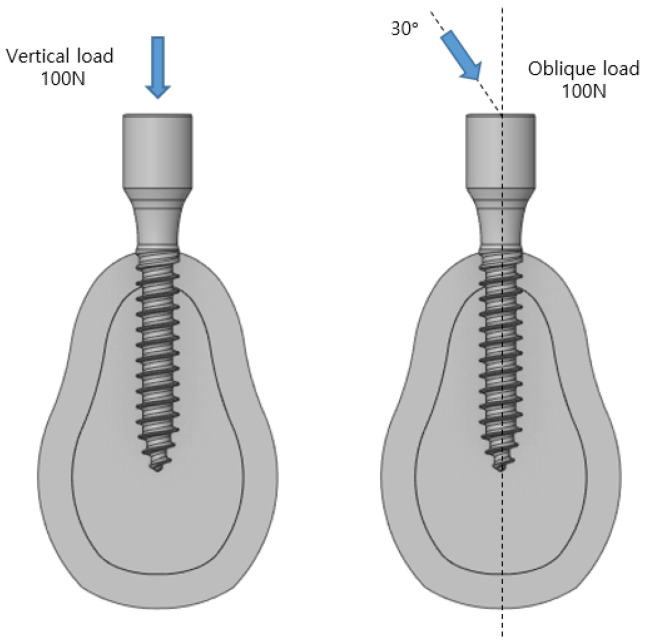
Axial, oblique loading condition.

The cancellous and cortical bones shared nodes, and the outer section of the cortical bone was constrained in the x-, y-, and z-directions using a fixed point. Friction contact, wherein friction exists between the implant and cartilage, was created to ensure that changes in the bone tissue can occur when the implant and bone are completely adhered. The friction coefficients of the two objects were 0.2. In this model, two loads were applied as shown in Figure 3. In load condition 1, a 100 N vertical load was applied to the center of the crown; in load condition 2, a 100 N inclined load was applied to the center of the crown at an angle of 30° in the narrow direction. The inclined load was modeled to act from the inside of the mouth to the outside, the orientation at which the chewing of the food occurs.

#### 2.1.3. Material Property

The general implant thread and abutment are made of titanium, and the bone is composed of hard cortical bone and soft cancellous bone. For structural analysis, Young’s modulus and Poisson’s ratios of the three materials are required. For this purpose, physical properties obtained through previous studies were used, as shown in Table 1 [16].

### 2.2. Analysis Method

#### 2.2.1. Analysis Based on Implant Length (Considering Load and Thickness Changes)

**Figure 4:**
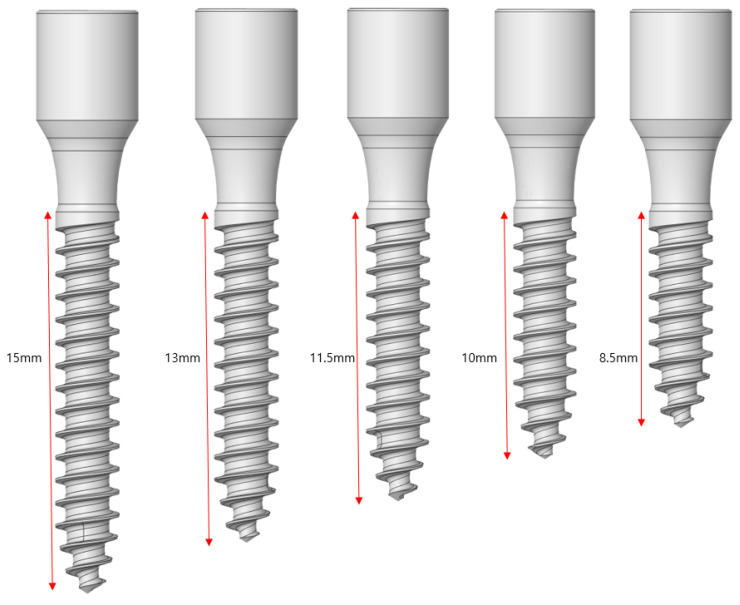
Implant length change.

An analysis was conducted by considering the length as an implant design variable. Five implant lengths were considered as shown in Figure 4: 15, 13, 11.5, 10, and 8.5 mm. A load was applied on 2.5- and 3.0 mm-thick samples along the vertical and oblique directions.

#### 2.2.2. Analysis Based on Physical Properties of Cancellous Bone (Considering Load and Thickness Changes)

An analysis was conducted by considering the physical properties of cancellous bone as design variables; the physical properties are listed in Table 2. A load was applied on 2.5- and 3.0 mm-thick samples along the vertical and oblique directions.

#### 2.2.3. Analysis Based on Cartilage Thickness (Considering Load Change)

**Figure 5:**
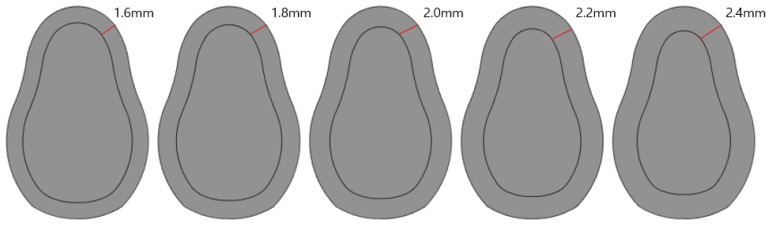
Cortical bone thickness.

An analysis was conducted by considering the cortical bone thickness as a design variable. Five cortical bone thicknesses were considered as shown in Figure 5: 1.6, 1.8, 2.0, 2.2, and 2.4 mm. Two types of loads were applied: vertical and oblique.

#### 2.2.4. Mesh Convergence Test

**Figure 6:**
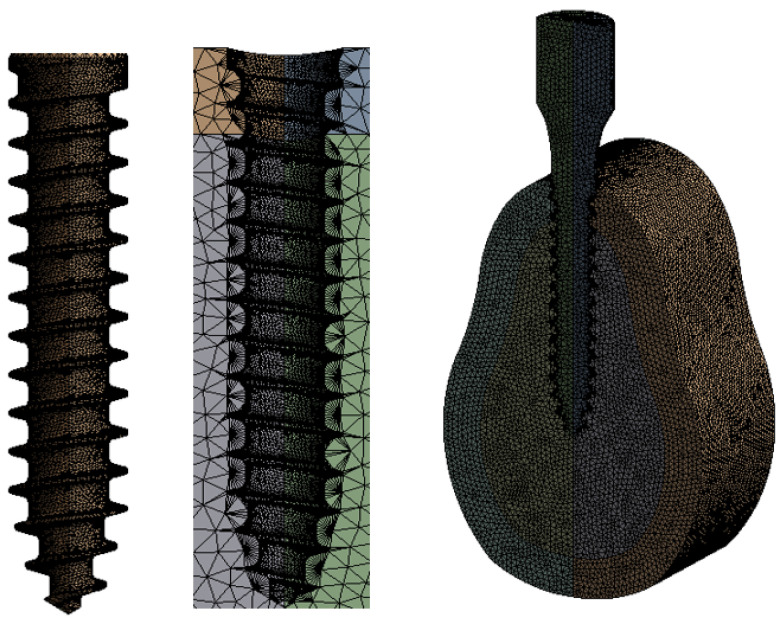
Implant, bone mesh.

**Figure 7:**
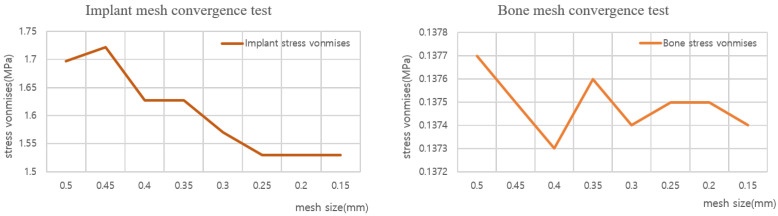
Chart of bone and implant mesh convergence test.

Each thread was 0.08 mm thick. Therefore, as shown in Figure 6 after setting the thread mesh size to which the contact was applied as 0.08 mm, convergence analysis was performed for the total body mesh. Table 3 shows the analysis results for the 0.05–0.5 mm range. The results converged from 0.25 mm; thus, the analysis was performed as depicted in Figure 7, with a mesh size of 0.25 mm and a thread size of 0.08 mm over the entire body.

## 3. Analysis Results and Considerations

### 3.1. Structural Analysis Results Based on Implant Length

**Figure 8:**
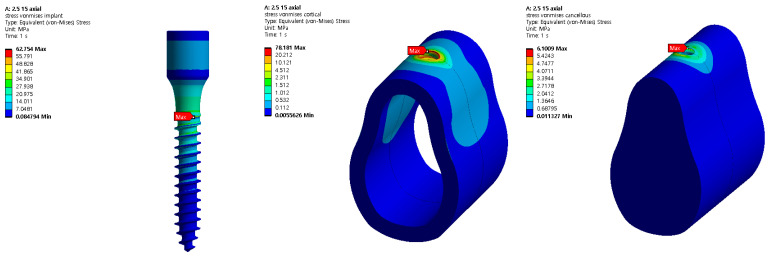
Von Mises stress analysis of the body with implant length 15 mm.

Structural analysis was performed using five different implant lengths under two load and two thickness conditions. For both the implants and bones, the stress value decreased from top to bottom. The highest stress was indicated on the first thread when both the implant and cortical bone were in contact with each other. Similarly, the highest stress was indicated at the first screw thread in contact with the cancellous bone, whereas a slight stress was indicated at the lower end of the bone hole, owing to contact with the implant. The analysis results for an implant length of 15 mm are shown in Figure 8. The stress generated in the oblique direction was approximately 10 times greater than that generated in the axial direction, and a high stress was indicated in the direction in which the force was applied.

**Figure 9:**
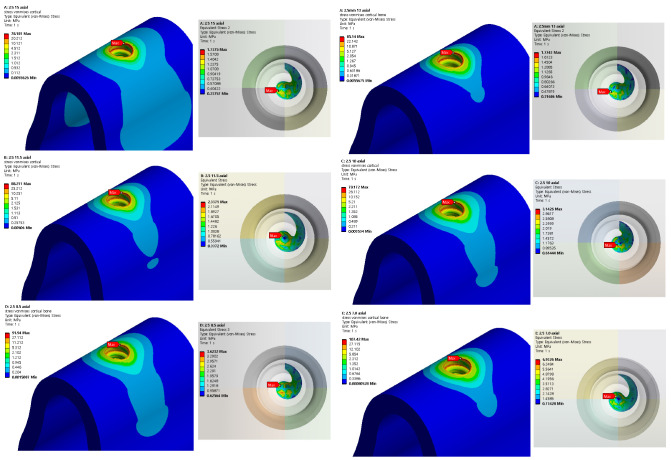
Von Mises stress on cortical bone, bottom of cancellous bone (2.5 mm).

**Figure 10:**
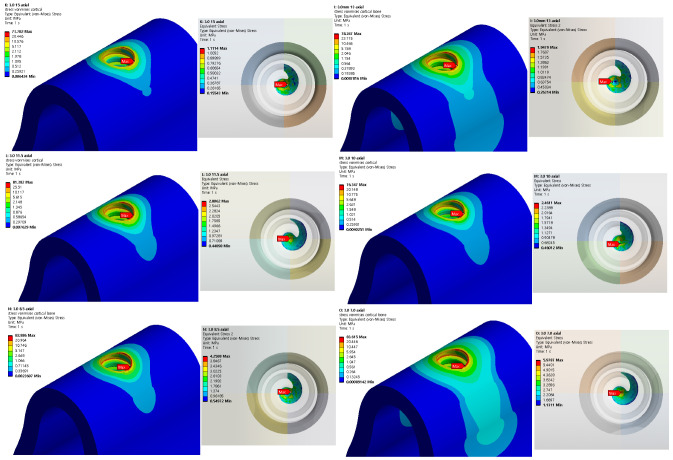
Von Mises stress on cortical bone, bottom of cancellous bone (3.0 mm).

Because titanium was used to construct the implant, plastic deformation did not occur below the maximum stress of 900 MPa in this analysis. In addition, because the cancellous bone does not contain nerves, it will not affect the human body, even if it is deformed. Accordingly, cortical bone stress values that can affect humans were evaluated. Figure 9 and Figure 10 show the results of cortical bone stress in each case, and stress values at the bottom of the cancellous bone hole that affect the stress of the cortical bone are summarized.

**Figure 11:**
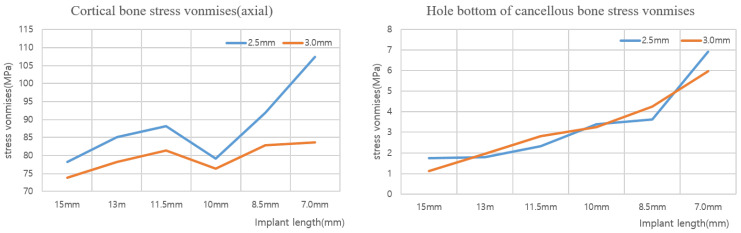
Cortical bone/hole in bone von Mises stress value charts (axial).

**Figure 12:**
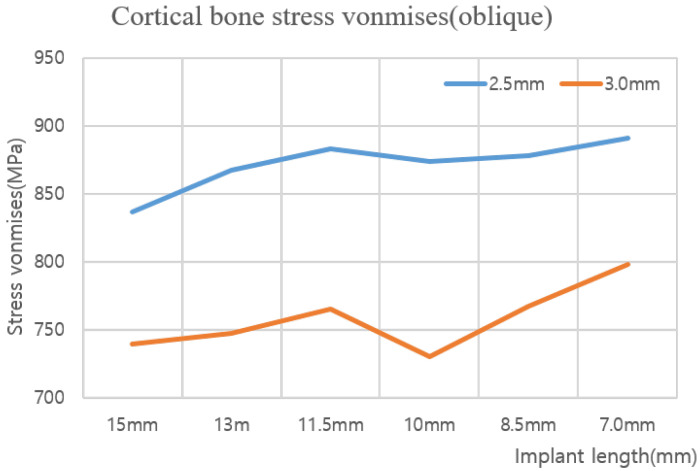
Cortical bone von Mises stress value charts (oblique).

Table 4 shows the maximum von Mises stress when an axial force with diameters of 2.5 and 3.0 mm is applied to the implant, and Figure 11 shows this as a chart. In addition, Table 5 shows the maximum von Mises stress when the oblique force of the implant diameter of 2.5 and 3.0 mm is applied, and Figure 12 shows this as a chart. Based on an analysis of the results, the stress generated in the cortical bone increased as the thickness decreased in both the axial and oblique directions. Meanwhile, based on the analysis results of implants reduced to 15, 13, and 11.5 mm, the von Mises stress increased gradually at the lower surface contact between the implant and cancellous bone but did not significantly affect the analysis results of the cortical bone. However, for the implant length of 10 mm, the von Mises stress on the lower surface increased, and the stress exerted on the cortical bone was dispersed. Thus, the cortical bone stress decreased. Even after that, although the level of stress distribution at the bottom of the hole in the bone increased, the length of the implant was greatly reduced, and the level of stress applied to the cortical bone showed a tendency to increase again with a high width. Meanwhile, when the implant thickness increased from 2.5 to 3.0 mm, the von Mises stress decreased in all cases. The best results were obtained when the implant thickness and length were set to 3.0 and 15 mm, respectively.

### 3.2. Structural Analysis Results Based on Cancellous Bone Elastic Modulus

**Figure 13:**
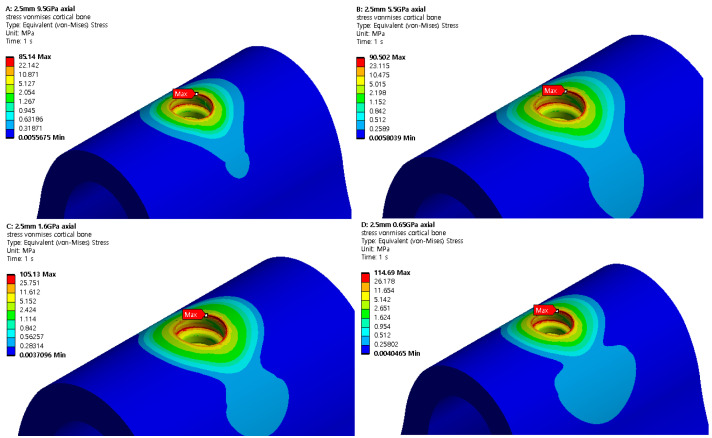
Cortical bone von Mises stress (2.5 mm).

**Figure 14:**
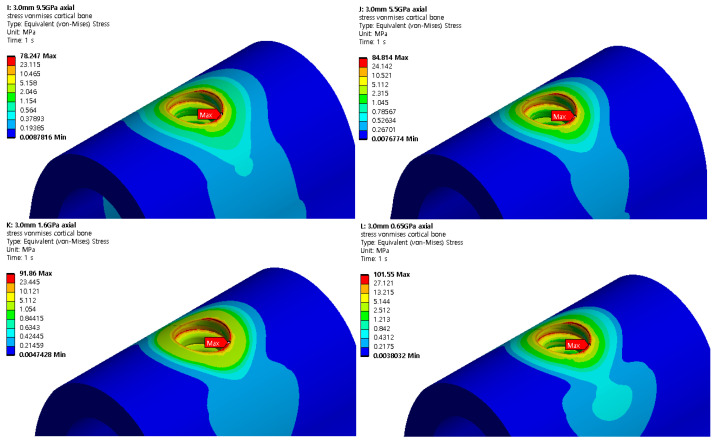
Cortical bone von Mises stress (3.0 mm).

**Figure 15:**
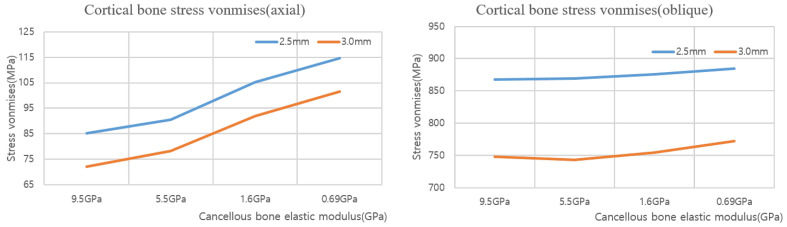
Cortical bone von Mises stress value charts.

A structural analysis was performed under two load and two thickness conditions using four cancellous bone types with different elastic moduli. The results are shown in Figure 13 and Figure 14. Table 6 and Table 7 show the maximum von Mises stresses of 2.5 and 3.0 mm implants when the elastic modulus of cancellous bone changes, and Figure 15 shows the results as a chart. The results show that the cortical bone stress increased as the cancellous bone elastic modulus decreased from 9.5 to 0.69 GPa in both the axial and oblique directions. When the elastic modulus of the cancellous bone decreased, the internal stress decreased because the bone became weaker. However, because the stress throughout the cartilage was relatively constant, the stress experienced by the cortical bone increased. Therefore, an implantation plan should be established for patients with a low cancellous bone coefficient of elasticity.

### 3.3. Structural Analysis Results Based on Cortical Bone Thickness

**Figure 16:**
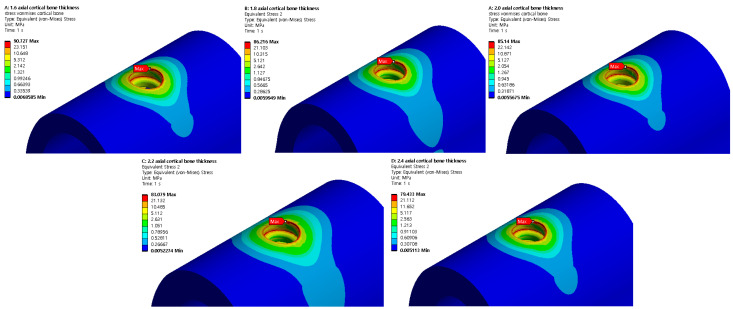
Cortical bone von Mises stress.

**Figure 17:**
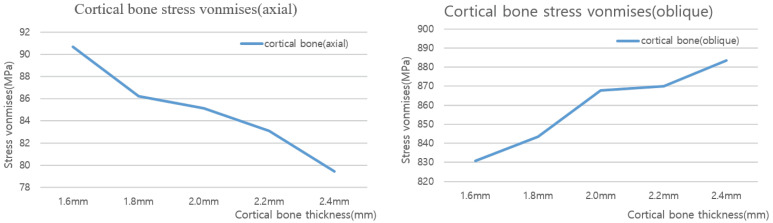
Cortical bone von Mises stress value charts.

Based on five different cortical bone thicknesses, structural analysis was performed under two load conditions. The results are shown in Figure 16. Table 8 shows the results of maximum von Mises stress according to the thickness of cortical bone, and Figure 17 shows the results as a chart. The stress decreased as the cortical bone thickness increased in the vertical direction; however, the stress increased in the oblique direction. In the vertical direction, a downward force was exerted on the thread; hence, the area of the force applied to the cortical bone increased as it became thicker, thereby reducing the stress. However, in the oblique direction, as the cortical bone became thicker, the stress generated in the cancellous bone due to the twisting of the cancellous bone dispersed to the cortical bone, thus resulting in a higher stress. Because the stress in the oblique direction was approximately nine times greater than that in the axial direction, an implantation plan should be established while considering greater stress values since the stress in the oblique direction was discovered to significantly affect a person with a thick cortical bone.

## 4. Discussion

In this study, the stress generated by the tooth and masticatory forces of an implant were analyzed via structural analysis using ANSYS. Implant length and thickness, cortical bone thickness, and cancellous bone elasticity modulus were used as design variables. In addition, the relationships among the design variables were analyzed.

The 3D model developed comprised two types of implants, i.e., those of the cortical bone and cancellous bone, and the implant and bone were assumed to be completely attached because the effect of the implant was analyzed after it was placed. The length and thickness of the implant used for a basic analysis were 13 and 2.5 mm, respectively. The implant was attached to the modeled cartilage, and a load of 100 N was applied in the vertical and oblique directions. The stress in the implant decreased from the upper to the lower section in the axial direction. In the oblique direction, a stress approximately 10 times that in the axial direction was generated in the opposite direction to which the force was applied. By comparing the maximum stress applied to the implant, its stability was determined based on the cortical bone, which affects the human body the most significantly because it does not undergo plastic deformation; furthermore, the cancellous bone does not contain any nerves and hence is not suitable for the analysis [17].

This is because the cortical and cancellous bones receive the same force simultaneously, and as the length decreases, the area that can be shared by the cancellous bone decreases. However, when the implant was 10 mm, the contact distance between the lower section of the cancellous bone and the implant reduced, and the stress increased because of an increase in the supporting force. As such, the stress on the 8.5 and 7.0 mm specimens was expected to reduce. However, compared with the increase in the force received from the bottom, the force in the contact area between the cancellous bone and implant decreased more significantly; therefore, the stress distribution increased. Therefore, as in the previous results, the stress generated in the cortical bone increased significantly. In addition, the stress increased by approximately nine times in the diagonal direction, and the basic trend was similar to that when the length decreased, i.e., the stress first increased, and after a brief pause at 10 mm, it increased. In addition, when the implant thickness increased to 3.0 mm, the maximum stress reduced because stress occurred in the cortical bone in a wider area compared with the case for the 2.5 mm-thick implant. Therefore, a long implant with a large area is advantageous to the human body. The results regarding the effects of implants according to diameter and length were compared using data comparing stability through resonance frequency analysis. Resonance frequency analysis is a bending force test for implants and bone assemblies. In this method, a constant lateral force is applied to the implant using magnetism, and the movement of the implant is measured. Peter [18] confirmed that the stability increases as the diameter increases, and Mats [19] confirmed that the failure rate increases as the implant length decreases. These results are consistent with the contents of this paper.

Lars [20] determined that bone density has a significant effect on implant failure rate. Bone strength varies depending on a person’s age, sex, and eating habits. In addition, the bone strength of a person may vary depending on the teeth position. For the case involving the cortical bone, no significant differences were observed since it is a normal bone; however, a significant difference was indicated in the cancellous bone because it is a cartilage. Therefore, the stability of the bones must be verified via analysis. A decrease in the elastic modulus of the cancellous bone indicates that the strength of the cartilage is weak. Analysis results show that the stress generated in the cancellous bone decreased as the cartilage became weaker. However, when the same force was applied, the force generated in the cortical bone increased because the amount of force generated on the entire bone was similar. Friberg [21] studied the lower failure rate of the mandible with high bone density compared to the upper jaw, obtaining results agreeing with those of this study.

Similarly, the thickness of the cortical bone may vary by person, even if the overall size of the bone is the same. Therefore, an analysis was performed by varying the thickness of the cortical bone to 1.6, 1.8, 2.0, 2.2, and 2.4 mm for the same bone size. The result shows that as the thickness increased, the stress decreased gradually when a vertical load was applied. However, when the load was applied in the oblique direction, the stress increased. However, the maximum stress generated by collision between teeth was 90 MPa, and the maximum amount of force generated by chewing food was 883.45 MPa, i.e., approximately 10 times greater. Therefore, the force generated when chewing food should be considered first. Additionally, a thinner cortical bone is more advantageous.

In this study, the characteristics of stress applied to the cortical bone were investigated based on four design conditions. In the future, we plan to extend our structural analysis by adding two more conditions: angle and implant position. In addition, after establishing a reduced-order model based on the results obtained, we plan to evaluate the optimal combination that reduces the probability of implantation failure using implantation parameters. The goal is to create an application that benefits both patients and doctors by loading them into implant placement programs to identify and monitor suitable implants in real time during implant placement. Using basic data obtained through digital dentistry will allow patients to not only observe the implantation process on a mobile device, but also to verify the stability of the implant after it is placed on them.

## 5. Conclusions

In this study, the stability based on force generated according to the implant length, radius, physical properties of cancellous bone, and thickness of external cartilage after implant placement was investigated. A longer implant length, larger radius, larger Young’s modulus of the cancellous bone, and thinner external cartilage are more advantageous because the level of stress applied to the cortical bone decreases. However, because each person has different bone characteristics, sizes of nearby teeth, and implantation angles, the conditions for implantation are different. Therefore, it is important to establish an implantation plan that meets the conditions rather than making a selection based on simple analysis data. In future research, the sensitivity of each variable will be analyzed using the data obtained through the analysis as a reduced-order model. This will make it possible to establish an automatic implantation plan using information obtained from people’s oral imaging information. In addition, this information will be applied to a mobile application to present an empirical basis for the doctor, and patients will be able to visually check their status, shorten the current complex process, and increase stability. 

## Figures and Tables

**Table 1:** Material properties.

Materials	Young’s Modulus (GPa)	Poisson’s Ratio
Implant	105	0.37
Cancellous bone	9.5	0.3
Cortical bone	13	0.3

**Table 2:** Cancellous bone material properties.

Materials	Young’s Modulus (GPa)	Poisson’s Ratio
Cancellous bone Type-1	9.5	0.3
Cancellous bone Type-2	5.5	0.3
Cancellous bone Type-3	1.6	0.3
Cancellous bone Type-4	0.69	0.3

**Table 3:** Mesh convergence test result.

	0.5	0.45	0.4	0.35	0.3	0.25	0.2	0.15
Implant	1.697	1.7219	1.6275	1.6273	1.5699	1.5299	1.5298	1.5297
Bone	0.13770	0.1375	0.1373	0.1376	0.1374	0.1375	0.1375	0.1374

**Table 4:** Cortical bone, hole in bone 2.5 mm/3.0 mm (axial force) von Mises stress values.

	15 mm	13 mm	11.5 mm	10 mm	8.5 mm	7.0 mm
Cortical bone (2.5 mm)	78.18 MPa	85.14 MPa	88.21 MPa	79.17 MPa	91.94 MPa	107.42 MPa
Hole of bone (2.5 mm)	1.74 MPa	1.78 MPa	2.33 MPa	3.14 MPa	3.62 MPa	6.93 MPa
Cortical bone (3.0 mm)	73.78 MPa	78.24 MPa	81.38 MPa	76.35 MPa	82.89 MPa	83.62 MPa
Hole of bone (3.0 mm)	1.11 MPa	1.95 MPa	2.81 MPa	3.26 MPa	4.26 MPa	5.98 MPa

**Table 5:** Cortical bone, Hole of bone 2.5 mm/3.0 mm (oblique force) von Mises stress values.

	15 mm	13 mm	11.5 mm	10 mm	8.5 mm	7.0 mm
Cortical bone (2.5 mm)	837.11 MPa	867.68 MPa	883.62 MPa	873.90 MPa	878.44 MPa	891.21 MPa
Cortical bone (3.0 mm)	739.82 MPa	747.57 MPa	765.54 MPa	730.62 MPa	767.42 MPa	798.15 MPa

**Table 6:** Cortical bone 2.5 mm/3.0 mm (axial force) von Mises stress values.

	9.5 GPa	5.5 GPa	1.6 GPa	0.69 GPa
2.5 mm	85.14 MPa	90.50 MPa	105.13 MPa	114.69 MPa
3.0 mm	72.12 MPa	78.25 MPa	91.86 MPa	101.55 MPa

**Table 7:** Cortical bone 2.5 mm/3.0 mm (oblique force) von Mises stress values.

	9.5 GPa	5.5 GPa	1.6 GPa	0.69 GPa
2.5 mm	867.68 MPa	868.83 MPa	875.52 MPa	884.20 MPa
3.0 mm	747.57 MPa	743.04 MPa	753.97 MPa	771.75 MPa

**Table 8:** Cortical bone von Mises stress values.

	1.6 mm	1.8 mm	2.0 mm	2.2 mm	2.4 mm
Axial	90.727 MPa	86.216 MPa	85.140 MPa	83.079 MPa	79.433 MPa
Oblique	830.89 MPa	843.39 MPa	867.68 MPa	870.1 MPa	883.45 MPa

## Data Availability

The data used to support the finding of this study are included within the article.
